# Bidirectional Relationship Between Myopia and Mental Disorders

**DOI:** 10.1155/da/3543589

**Published:** 2025-12-01

**Authors:** Dan-Lin Li, Su-Tong Yu, Li-Jun Zhang, Xiao-Feng Zhang, Chen-Wei Pan

**Affiliations:** ^1^School of Public Health, Suzhou Medical College of Soochow University, Suzhou, China; ^2^Suzhou Medical College of Soochow University, Suzhou, China; ^3^Department of Ophthalmology, Dalian Third People's Hospital Affiliated to Dalian University of Technology, Dalian, China; ^4^Department of Ophthalmology, The Fourth Affiliated Hospital of Soochow University, Suzhou, China

**Keywords:** bidirectional relationship, mental disorders, myopia

## Abstract

**Background:**

Myopia and mental disorders share common environmental and behavioral risk factors. Previous studies have been inconsistent regarding the association between them, this systematic review and meta-analysis aims to explore the association between myopia and mental disorders.

**Methods:**

Databases, including PubMed, Web of Science, Wiley online library, and Cochrane Library were searched for eligible publications from January 2000 to July 2024. Studies assessing the relationship between myopia and any one of the mental disorders were identified. Pooled odds ratios (ORs) and their corresponding 95% confidence intervals (95% CIs) were estimated using random-effects models.

**Results:**

We identified 15 articles (two cohort studies and 13 cross-sectional studies) examining the impact of myopia on mental disorders, involving 2,841,653 participants from seven countries. Pooled results indicated that myopia was significantly associated with an increased risk of mental disorders (OR = 1.41, 95% CI: 1.23, 1.59). Besides, a significant relationship was observed between the mental disorders and myopia (OR = 1.15, 95% CI: 1.01, 1.29) based on eight articles, including 1,942,855 participants from five countries. Subgroup analyses showed that the association of myopia with both anxiety and depression was significant, with ORs of 1.49 and 1.33, respectively.

**Conclusions:**

Myopia and mental disorders may influence each other, with each condition potentially exacerbating the risk of the other. These findings help to explore the possible interplay between mental disorders and myopia.

## 1. Introduction

In recent years, the prevalence of both myopia and mental disorders has increased significantly [1, 2]. This trend has also sparked increased interest in exploring the potential associations between myopia and mental health. Prior research has indicated a complex and interdependent association between these two disorders, manifested through shared risk factors (e.g., academic performance and socioeconomic status), common pathophysiological pathways (e.g., chronic inflammation and genetic susceptibility), and even a potential causal link demonstrating temporal precedence and elevated incident risk of one condition following the other [3–5]. Some studies suggested that myopia may be associated with an elevated risk of mental disorders, such as anxiety and depression [6–8]. Conversely, psychological factors, including stress and anxiety, have been implicated in the etiology and progression of myopia [9, 10].

Despite this emerging body of evidence, the relationship between myopia and mental health remains incompletely understood. Some studies suggest a more pronounced connection between myopia and anxiety, while a link with depression may not exist [11, 12]. Additionally, the association is inconsistent across populations, being more common in children and adolescents [13]. Previous research into the correlation between myopia and mental health has focused mainly on targeted groups or specific mental health outcomes, without a comprehensive and integrated consideration of their potential interaction. Although preliminary findings suggest a possible bidirectional association between myopia and mental disorders, robust evidence confirming this relationship remains insufficient [3]. Therefore, further investigation utilizing higher levels of evidence is necessary to clarify this association.

Given the significant clinical and public health implications, exploring the relationship between myopia and mental disorders is critical for understanding their interrelationship and mechanisms. Therefore, this study undertakes a comprehensive synthesis of existing primary research to quantitatively evaluate the effect of myopia on mental health and the reverse.

## 2. Methods

This systematic review and meta-analysis were conducted in accordance with the meta-analysis of observational studies in epidemiology (MOOSE) guidelines [14]. The protocol for this study was registered in the International Prospective Register of Systematic Reviews (PROSPERO) with the registration number CRD42024579354. The entire process of literature search, selection, data extraction, and risk of bias assessment was independently performed by two reviewers. Any discrepancies were resolved through discussion with a third reviewer. The Kappa coefficient for inter-reviewer agreement in each step exceeded 0.85, indicating a high level of consistency.

### 2.1. Search Strategy

A comprehensive search for relevant literature was conducted across PubMed, Web of Science, Wiley Online Library, and Cochrane Library from their inception until July 18, 2024. The search strategy employed Medical Subject Headings (MeSH) terms and free-text words in combination with various keywords related to myopia (e.g., “myopia [MeSH Terms],” “myopia [Title/Abstract],” “refractive error [MeSH Terms],” and “nearsightedness [Title/Abstract]”) and mental disorders (e.g., “mental disorders [MeSH Terms],” “mental health [Title/Abstract],” “psychopathology [Title/Abstract],” and “emotion [Title/Abstract]”). The search queries were tailored to the specific syntactic requirements of each database. The complete search strategies are presented in Supporting Information 1: Table S1. Furthermore, reference lists of identified papers were also screened manually to minimize the chance of missing studies that are relevant.

### 2.2. Eligibility Criteria and Data Extraction

All retrieved articles were imported into EndNote 21 for management and screening. Studies were included based on the following criteria: (a) population-based cross-sectional or cohort studies; (b) published in English; (c) reporting an association between mental disorders and myopia; (d) providing sufficient data to calculate odds ratios (ORs) and 95% confidence intervals (CIs); (e) in situations involving overlapping study populations with the same reported outcome, the study with the larger sample size was selected. Studies were excluded if they were: (a) systematic reviews, meta-analyses, editorials, case reports, comments, or letters; (b) conference abstracts; (c) conducted on animals or in vitro. Data extracted from each study included the first author's name, publication year, country of origin, study design, participant age, sample size, myopia assessment method, type of mental disorder and its assessment method, and controlled variables.

### 2.3. Quality Assessment

The quality of included cohort studies was evaluated using the Newcastle–Ottawa Quality Scale (NOS). The scale assesses research on three axes: selection of the study group, comparability of groups, and ascertainment of the outcome of interest. 7–9 were of high quality, 4–6 of moderate quality, and 3 or less of low quality [15].

For cross-sectional studies, the Agency for Healthcare Research and Quality (AHRQ) criteria were used for quality assessment. This criteria consists of 11 items, each scored as “1” for “yes” and “0” for “no” or “unclear.” Studies with total scores ranging from 0 to 3 were considered low quality, 4–7 as medium quality, and 8–11 as high quality [16]. Studies rated as low quality were excluded from the analysis.

### 2.4. Statistical Analyses

In our study, mental disorders and myopia were analyzed as separate endpoints. All included studies contributed exactly one independent effect estimate to the meta-analysis. When multiple ORs were reported within a single study, we implemented a structured procedure: (1) when both crude and adjusted ORs were available, the estimate from the most fully adjusted model was selected; (2) for studies reporting stratified results (e.g., sex and specific psychological measures), subgroup-specific ORs were pooled into a single summary estimate; (3) studies reporting only one OR were included directly. This processing ensured statistical robustness, prevented within-study overrepresentation in the subsequent meta-analysis.

Heterogeneity among included studies was assessed using the *I*^2^ statistic. An *I*^2^ value < 50% indicated low heterogeneity, allowing the use of a fixed-effects model for combined analysis. Conversely, an *I*^2^ value ≥ 50% indicated significant heterogeneity, necessitating the use of a random-effects model. Subgroup analyses were conducted to explore potential sources of heterogeneity. Sensitivity analyses were performed by sequentially removing one study to test the robustness of the results. Additionally, publication bias was assessed using Egger's test, with a *p*-value < 0.05 considered statistically significant. All statistical analyses were conducted using Stata 18.0 software.

## 3. Results

**Figure 1:**
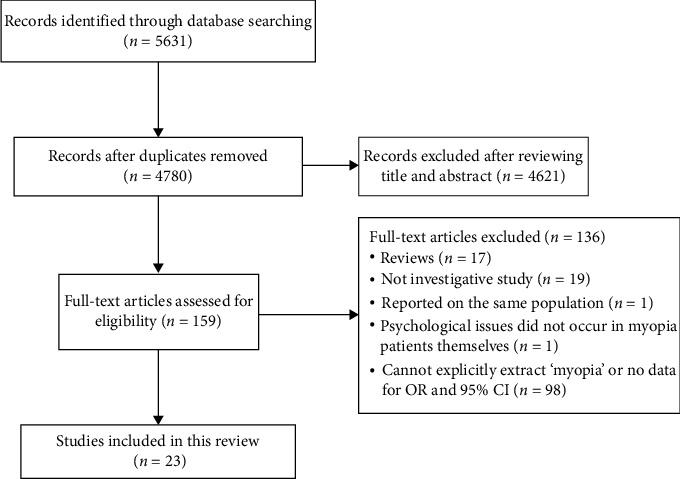
Selection process of articles review.

An initial database search identified a total of 4780 documents, with duplicates removed (Figure 1). Upon meticulous examination of the titles and abstracts, 4621 records were deemed ineligible and excluded, leaving 159 full-text articles for further screening. Ultimately, 23 studies fulfilled the inclusion and exclusion criteria, categorizing into two main groups: 15 studies examining the impact of myopia on mental disorders [3, 5, 7, 8, 11, 17–26] and eight studies exploring the impact of mental disorders on myopia [3, 9, 10, 27–31]. However, during subsequent analyses, two studies [3, 26] exhibited significant heterogeneity, and one study [19] demonstrated publication bias. Consequently, a total of 12 articles were included in the final meta-analysis.

### 3.1. Study Characteristics on Myopia's Impact on Mental Disorders

The review of the impact of myopia on mental disorders incorporated 15 articles (two cohort studies and 13 cross-sectional studies). The sample sizes ranged widely, from 94 to 1,884,701 participants, summing to a total of 2,841,653 individuals, encompassing both children and adults aged 5 years and older. The studies were published between 2008 and 2024, with geographical representation from China (nine studies), Israel, Ghana, Poland, Japan, Singapore, and Australia (one study each).

Mental disorders considered were depressive symptoms, anxiety, mood disorders, attention-deficit/hyperactivity disorder (ADHD), psychological distress, cognitive dysfunction, and personality disorders. Diverse rating questionnaires and assessment methodologies were utilized to evaluate these psychological conditions, with detailed characteristics outlined in Table 1. Quality assessments revealed that three studies were of moderate quality, while the remaining studies were of high quality, as specified in Supporting Information 1: Table S2.

### 3.2. Study Characteristics on Mental Disorders' Impact on Myopia

A total of eight studies (two cohort studies and six cross-sectional studies) were included in the synthesis of the effect of mental disorders on myopia. The combined sample size ranged from 100 to 1,884,701 participants, totaling 1,942,855 individuals aged 1–36 years. These studies were published between 2008 and 2024, with contributions from China (four studies), Spain, Germany, the United Kingdom, and Turkey (one study each).

The mental disorders explored encompassed ADHD, general mental disorders, autism spectrum disorder (ASD), stress, and intellectual disability. Notably, all studies except one were based on clinical diagnoses; the exception was a self-reported study. The specific characteristics of included studies are presented in Table 2. In Supporting Information 1: Table S3, quality assessments results indicated that three studies were of high quality, and five were of moderate quality.

### 3.3. Quantitative Synthesis of Results

**Figure 2:**
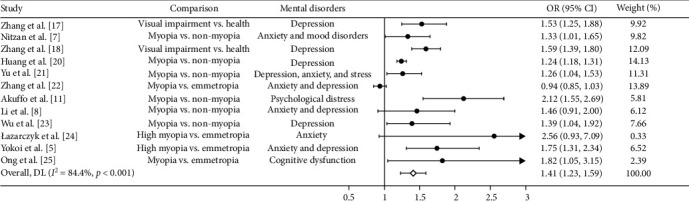
Forest plot of the associations of myopia or high myopia with mental disorders. CI, confidence interval; OR, odds ratio.

**Figure 3:**
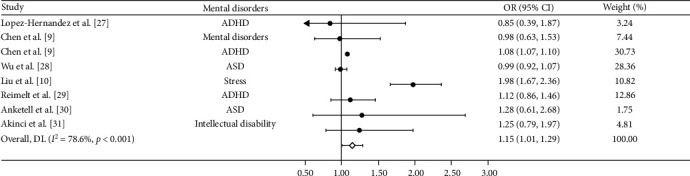
Forest plot of the associations of mental disorders with myopia. CI, confidence interval; OR, odds ratio.

Based on sensitivity analysis, we excluded three of the 15 studies from the pooled analysis because of a high or unclear risk of attrition bias, which left us with the final 12 studies for quantitative synthesis. The forest plot depicted in Figure 2 illustrates a statistically significant correlation between individuals with myopia or high myopia and the occurrence of mental disorders (OR = 1.41, 95% CI: 1.23–1.59), when compared to those without myopia or who are emmetropic. The *I*^2^ statistic value for the heterogeneity results is 84.4%. In addition, forest plots investigating the effect of mental disorders on myopia revealed a significant effect, indicating that mental disorders are associated with an increased risk of developing myopia (OR = 1.15, 95% CI: 1.01–1.29; Figure 3). The *I*^2^ statistic for this particular analysis was 78.6%.

Sensitivity analysis charts for both meta-analyses were generated to ensure the robustness of the combined results. As depicted in Supporting Information 1: Figures S1 and S2, the results were found to be stable and reliable. Besides, Egger's test was employed to reveal any potential publication bias, and no significant bias was detected for all the outcomes (all *p*-values for Egger's test > 0.05).

### 3.4. Subgroup Analysis

**Figure 4:**
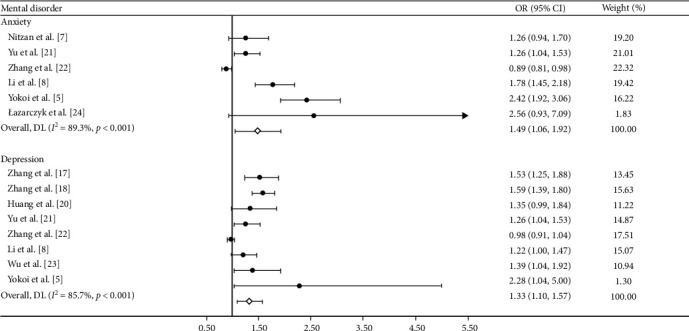
Forest plot of the associations of myopia or high myopia with depression and anxiety. CI, confidence interval; OR, odds ratio.

To further examine the association of myopia with some mental health outcomes, subgroup analyses for depression and anxiety were performed. These analyses revealed that myopia/high myopia was significantly associated with both anxiety (OR = 1.49, 95% CI: 1.06–1.92) and depression (OR = 1.33, 95% CI: 1.10–1.57), as shown in Figure 4.

To assess the impact on the associations between sample size, we performed subgroup analyses using a cut point of 5000 participants. The forest plots in Supporting Information 2: Figure S3 indicate that myopia–high myopia was significantly associated with mental disorders in the studies having sample sizes greater than 5000 (OR: 1.44, 95% CI: 1.25–1.63). Similarly, a significant association was observed in studies with a sample size of less than 5000 (OR = 1.41, 95% CI: 1.00–1.81). The results from the forest plots (Supporting Information 2: Figure S4) revealed a nonsignificant association of mental disorders on myopia/high myopia in the subgroup with sample sizes exceeding 5000 (OR = 1.05, 95% CI: 0.97–1.12). And in studies with smaller sample sizes (<5000), the result also was not statistically significant (OR = 1.35, 95% CI: 0.73–1.98). These findings indicate that sample size is unlikely to be a source of heterogeneity in the meta-analysis.

Additionally, subgroup analyses were conducted based on age (<30 years vs. ≥30 years). As detailed in Supporting Information 2: Figures S5 and S6, in the analysis assessing the effect of myopia on subsequent mental disorders, the pooled OR was 1.31 (95% CI: 1.12–1.50) for populations with a mean age under 30 years, and 1.72 (95% CI: 1.38–2.06) for those aged 30 years or older. For mental disorder comorbidity as a risk indicator for myopia, population data below the age of 30 years were only available for study, and this yielded a significant OR of 1.08 (95% CI: 1.06–1.09). These results suggest that age does not serve as a source of heterogeneity between myopia and disorders.

## 4. Discussion

The present systematic review and meta-analysis revealed intriguing findings regarding the relationship between myopia and mental disorders. By pooling data from 23 epidemiological studies, which included both cross-sectional and longitudinal designs, we found a significant association between myopia and various mental health issues, such as depressive symptoms, anxiety, and psychological distress. Specifically, individuals with myopia were more likely to experience these mental disorders compared to those without. Conversely, mental disorders also showed an association with increased risk of development of myopia, suggesting a bidirectional relationship between the two conditions.

At the sociocultural level, modern lifestyles, with increased digital device usage and prolonged near work activities, have been implicated in the etiology of myopia [32]. Competitive education and work systems expose both adolescents and adults to various risks of mental health issues. These pressures of study and environment may also exacerbate psychological stress and anxiety, thereby contributing to the development of mental disorders [8, 33]. In this context, prolonged periods of concentration and reduced opportunities for rest and relaxation have become common risk factors for both myopia and psychological health [34, 35]. Individual behavioral factors also play a crucial role in the relationship between myopia and psychological health [36, 37]. Similarly, individuals with certain behavioral traits, such as perfectionism or a tendency to ruminate over problems, may be more susceptible to psychological stress and anxiety [38]. In addition, students with myopia who wear glasses may become the target of bullying due to their visual impairment in social situations, which can lead to psychosocial maladjustment and emotional issues [8]. These behavioral habits can also strengthen the two-way relationship of myopia with psychiatric disorders, creating a vicious cycle of poor eye and mental health.

In the physiological perspective, myopia and psychological health share common pathways. Research indicates a correlation between psychological stress-induced events and pseudo-myopia [39]. Psychological events affect the autonomic nervous system, causing the ciliary muscle to contract in response to anxiety, which in turn can lead to symptoms of myopia [39]. This viewpoint aligns with another study's conclusion that myopia is a result of a defense mechanism against tension. This tension causes the extraocular muscles around the eyeball to tighten, directly leading to refractive errors [40]. Moreover, sunlight not only helps prevent myopia but also serves as a “medicine” for various clinical conditions, including depression and sleep disorders, where vitamin D levels and metabolism play a significant role [41]. Insufficient sunlight, a hallmark of modern indoor lifestyles, can disrupt circadian rhythms [34]. This disruption not only lowers retinal dopamine levels—a critical stop signal for axial elongation of the eye—but also serves as a well-established factor in the pathogenesis of mood disorders [41, 42]. Furthermore, chronic psychological stress can trigger a state of low-grade systemic inflammation [19]. The resulting proinflammatory cytokines may compromise the blood-retinal barrier, promoting myopia progression, while simultaneously acting on the central nervous system to exacerbate anxiety and depressive behaviors [43].

From a genetic perspective, there is evidence to suggesting that myopia and psychiatric disorders have not only their own genetic components but also share common genetic factors and protein expressions [44–46]. Research by Puranik and Song [44] has summarized that variations in the expression of Slitrk proteins are closely associated with both myopia and neuropsychiatric conditions. However, current research on these two conditions often focuses on specific clinical diseases, such as Cohen syndrome, or developmental abnormalities, with relatively less research on emotional psychological disorders [47, 48]. To a large extent, both psychology and myopia are significantly influenced by environmental factors. Still, it is not impossible that individuals with a genetic predisposition for myopia would be particularly susceptible to environmental stress, causing an enhancement in eye strain caused by near work. Once more, individuals with a gene risk for mental illness might be particularly susceptible to the psychological implications of visual impairments. The interplay between genetic factors and environmental influences could further complicate the relationship between myopia and mental health.

The three studies excluded from this meta-analysis exhibit distinct methodological features that may explain their heterogeneity or risk of bias. Chou et al. [3] reported a bidirectional relationship between ADHD and myopia using a Taiwanese health claims database. Their retrospective design may precipitate unmeasured confounding due to geographical variation in healthcare access or diagnostic practice, which may account for the observed heterogeneity. van de Berg et al. [26] found no strong evidence for a “myopic personality,” and their findings, based on a sample of twins and clinic-based families with a broad myopia definition (≤−0.50 D), may have limited generalizability. Finally, Zhu et al. [19] focused specifically on high myopia (axial length 26 mm) and its link with anxiety via CCL2-induced inflammation—a mechanistic focus far afield from the epidemiological scope of the other trials covered here, and highly likely to create publication bias clues.

While this study offers a strong assessment of bidirectional association between myopia and mental disorders, it does so subject to certain constraints. First, only one study examined the bidirectional relationship within the same population [3], highlighting a gap in the current evidence base. Second, heterogeneity in primary study designs and assessment methods for both myopia and mental disorders may have variability into the pooled estimates. Third, the causal interpretation of the association is limited by the predominantly cross-sectional design of included studies. While a bidirectional association is supported, the inability to infer causality remains a key constraint. Finally, given the substantial diversity in the composition of the included studies across countries, sociocultural backgrounds, healthcare systems, and participant age ranges, it is possible that unmeasured confounding factors have influenced the results, thereby affecting their generalizability. Future work needs to employ longitudinal and interventional designs to elucidate causal mechanisms between these conditions.

The clinical and public health implications of our findings are significant. Given the bidirectional relationship between myopia and mental disorders, addressing one condition may have beneficial effects on the other. For instance, interventions that limit screen time and promote exercise outdoors, known to slow myopia progression, also have the potential to positively impact mental health. Conversely, treating mental disorders, such as anxiety and depression, may help in managing the progression of myopia. These findings emphasize the need for comprehensive and integrated approaches to address both ocular and mental health issues.

## 5. Conclusion

This meta-analysis provides further evidence for a significant association between myopia and mental disorders. The findings provide valuable insight into the dynamic relationship between myopia and mental health, with implications for the clinical relevance of developing combined interventions for managing both conditions together. Future research should explore the underlying mechanisms of this association, including sociocultural, individual behavioral, and genetic factors, to inform the development of more targeted and effective interventions.

## Figures and Tables

**Table 1:** Characteristics of the included literature of the impact of myopia on mental disorders (*n* = 15).

Author, year	Country	Design	Age (years)	Myopia assessment	Comparison	Sample size	Mental disorders	Mental disorders assessment	OR (95% CI)	Quality assessment
Zhang et al., 2024 [17]	China	Cohort	12.1 ± 2.5	Visual impairment (standard logarithmic visual acuity chart)	Visual impairment vs. health	15,348	Depressive symptoms	CES-D	1.53 (1.25, 1.88)	High
Nitzan et al., 2024 [7]	Israel	Cross-sectional	17.2 ± 0.7	SEQ (noncycloplegic)	Myopia vs. non-myopia	891,501	Anxiety and mood disorders	Psychiatric consultant and ICD-10	1.33 (1.01, 1.65)	High
Zhang et al., 2023 [18]	China	Cross-sectional	14.4 ± 1.7	Visual impairment (standard logarithmic visual acuity chart)	Visual impairment vs. health	8997	Depressive symptoms	CES-D	1.59 (1.39, 1.80)	High
Chou et al., 2023 [3]^a^	China	Cohort	5–14	Disease code	Myopia vs. non-myopia	1,884,701	ADHD	Clinical diagnostic	1.07 (1.05, 1.09)	High
Zhu et al., 2023 [19]^a^	China	Cross-sectional	Unclear	AL (high myopia: AL ≥ 26 mm; emmetropia: AL 22–24.5 mm)	High myopia vs. emmetropia	210	Anxiety	HAMA	8.69 (2.50, 30.12)	Medium
Huang et al., 2022 [20]	China	Cross-sectional	14.11 ± 1.25	Self-report	Myopia vs. non-myopia	19,299	Depression	CES-D	1.24 (1.18, 1.31)	Medium
Yu et al., 2022 [21]	China	Cross-sectional	19.82 ± 1.43	Unclear	Myopia vs. non-myopia	6032	Depression, anxiety, stress	DASS-21	1.26 (1.04, 1.53)	High
Zhang et al., 2021 [22]	China	Cross-sectional	18.2 ± 0.7	SE (noncycloplegic)	Myopia vs. emmetropia	764	Anxiety, depression	SAS/SDS	0.94 (0.85, 1.03)	High
Akuffo et al., 2021 [11]	Ghana	Cross-sectional	38.4 ± 0.35	Self-report	Myopia vs. non-myopia	6859	Psychological distress	K10	2.12 (1.55, 2.69)	High
Li et al., 2020 [8]	China	Cross-sectional	14–17	SE (cycloplegic)	Myopia vs. non-myopia	1103	Anxiety, depression	SAS/SDS	1.46 (0.91,2.00)	High
Wu et al., 2017 [23]	China	Cross-sectional	> 60	SE (objective refraction/autorefraction)	Myopia vs. Non-myopia	4597	Depressive symptoms	PHQ-9	1.39 (1.04, 1.92)	High
Łazarczyk et al., 2016 [24]	Poland	Cross-sectional	13–14	SE	High myopia vs. emmetropia	94	Anxiety	STAIC	2.56 (0.93, 7.09)	High
Yokoi et al., 2014 [5]	Japan	Cross-sectional	45–68	SE/AL	High myopia vs. emmetropia	205	Anxiety and depression	HADS	1.75 (1.31, 2.34)	High
Ong et al., 2013 [25]	Singapore	Cross-sectional	60–79	SE (noncycloplegic)	Myopia vs. emmetropia	1032	Cognitive dysfunction	AMT	1.82 (1.05, 3.15)	High
van de Berg et al., 2008 [26]^a^	Australia	Cross-sectional Study	53.04	SE (dilated autorefraction)	Myopia vs. non-myopia	911	Personality	IPIP five factor inventory	1.00 (0.99, 1.01)	Medium

*Note*: DASS-21, 21-item depression, anxiety, and stress scale; K10, Kessler psychological distress scale; PHQ-9, 9-item Patient Health Questionnaire.

Abbreviations: ADHD, attention-deficit/hyperactivity disorder; AL, axial length; AMT, abbreviated mental test; CES-D, Center for Epidemiologic Studies Depression; CI, confidence interval; HADS, hospital anxiety and depression scale; HAMA, Hamilton anxiety scale; ICD-10, 10-item International Classification of Diseases; IPIP, International Personality Item Pool; OR, odds ratio; SAS, self-rating anxiety scale; SDS, self-rating depression scale; SE, spherical equivalent; SEQ, spherical equivalent; STAIC, State-Trait Anxiety Inventory for Children.

^a^Articles are those not included in the meta-analysis.

**Table 2:** Characteristics of the included literature of the impact of mental disorders on myopia (*n* = 8).

Author, year	Country	Design	Age (years)	Mental disorders	Mental disorders assessment	Sample size	Myopia assessment	OR (95% CI)	Quality assessment
Lopez-Hernandez et al., 2024 [27]	Spain	Cross-sectional	6–36	ADHD	DSM-5 code	100	SE (noncycloplegic)	0.85 (0.39, 1.87)	Medium
Chen et al., 2023 [9]	China	Cross-sectional	6–18	Mental disorders	Clinical diagnostic	378	SE (noncycloplegic/cycloplegic)	0.98 (0.63, 1.53)	Medium
Chou et al., 2023 [3]	China	Cohort	5–14	ADHD	Clinical diagnostic	1,884,701	Disease code	1.08 (1.07, 1.10)	High
Wu et al., 2023 [28]	China	Cohort	≤18	ASD	ICD-9-CM code	39,061	Disease code	0.99 (0.92, 1.07)	High
Liu et al., 2021 [10]	China	Cross-sectional	Unclear	Stress	Self-report	3918	Self-report	1.98 (1.67, 2.36)	Medium
Reimelt et al., 2021 [29]	Germany	Cross-sectional	3–17	ADHD	Parental statement and SDQ	13,488	Parent report	1.12 (0.86, 1.46)	High
Anketell et al., 2016 [30]	UK	Cross-sectional	6–16	ASD	ICD code	334	SE (cycloplegic)	1.28 (0.61, 2.68)	Medium
Akinci et al., 2008 [31]	Turkey	Cross-sectional	1–17	Intellectual disability	Standardized formal IQ tests	875	SE (cycloplegic)	1.25 (0.79, 1.97)	Medium

Abbreviations: ADHD, attention-deficit/hyperactivity disorder; DSM-5, Diagnostic and Statistical Manual of Mental Disorders 5^th^ edition; ICD-9-CM, International Classification of Diseases; Ninth Revision, Clinical Modification; SDQ, strengths and difficulties questionnaire; SE, spherical equivalent.

## Data Availability

Data sharing is not applicable to this article as no new data were created or analyzed in this study.

## Supporting Information

Supporting Information 1 Table S1. Search strategy for identifying studies on the association between mental disorders and myopia. Table S2. The specific results of quality assessment of include articles of the impact of myopia on mental disorders (*n* = 15). Table S3. The specific results of quality assessment of include articles of the impact of mental disorders on myopia (*n* = 8).

Supporting Information 2 Figure S1. The sensitivity analyses of 12 articles on myopia affecting the mental disorders. Figure S2. The sensitivity analyses of eight articles on mental disorders affecting myopia. Figure S3. The sensitivity analyses of myopia on mental disorders by sample size. Figure S4. The sensitivity analyses of mental disorders on myopia by sample size. Figure S5. The sensitivity analyses of myopia on mental disorders by age. Figure S6. The analyses of mental disorders on myopia in those age under 30 years.

## References

[1] Rami F. Z., Li L., Le T. H., Kang C., Han M. A., Chung Y.-C. (2024). Risk and Protective Factors for Severe Mental Disorders in Asia. *Neuroscience and Biobehavioral Reviews*.

[2] Morgan I. G., Wu P. C., Ostrin L. A. (2021). IMI Risk Factors for Myopia. *Investigative Ophthalmology and Visual Science*.

[3] Chou W. P., Chen Y. L., Hsiao R. C., Lai Y. H., Yen C. F. (2023). Bidirectional Associations Between Hyperopia, Myopia, Astigmatism, and Strabismus, and Attention-Deficit/Hyperactivity Disorder in Children: A National Population-Based Cohort Study. *Brazilian Journal of Psychiatry*.

[4] Li D., Chan V. F., Virgili G. (2022). Impact of Vision Impairment and Ocular Morbidity and Their Treatment on Depression and Anxiety in Children: A Systematic Review. *Ophthalmology*.

[5] Yokoi T., Moriyama M., Hayashi K. (2014). Predictive Factors for Comorbid Psychiatric Disorders and Their Impact On Vision-Related Quality of Life in Patients With High Myopia. *International Ophthalmology*.

[6] Shu Q., Xiao Z., Peng X. (2024). Influencing Factors for Pediatric Eye Disorders and Health Related Quality of Life: A Cross-Sectional Study in Shanghai. *Frontiers In Medicine*.

[7] Nitzan I., Shmueli O., Safir M. (2024). Association of Myopia With Anxiety and Mood Disorders in Adolescents. *Eye*.

[8] Li Q., Yang J., He Y. (2020). Investigation of the Psychological Health of First-Year High School Students With Myopia in Guangzhou. *Brain and Behavior*.

[9] Chen L., Sun L., Xue C. (2023). Refractive Errors and Ocular Findings in Children and Adolescents With Mental Disorders: A Retrospective Study. *BMC Ophthalmology*.

[10] Liu J., Chen Q., Dang J. (2021). Examining Risk Factors Related to Digital Learning and Social Isolation: Youth Visual Acuity in COVID-19 Pandemic. *Journal of Global Health*.

[11] Akuffo K. O., Sewpaul R., Darrah S. (2021). Vision Difficulty and Psychological Distress in South Africa: Results From SANHANES-1. *BMC Psychology*.

[12] Li Z., Wei J., Lu S., Wang F., Xia Y. (2023). Association Between Myopia and Anxiety: A Cross-Sectional Study Based On Chinese University Freshmen. *Annals of Translational Medicine*.

[13] Ayaki M., Torii H., Tsubota K., Negishi K. (2016). Decreased Sleep Quality in High Myopia Children. *Scientific Reports*.

[14] Stroup D. F., Berlin J. A., Morton S. C. (2000). Meta-Analysis of Observational Studies in Epidemiology: A Proposal for Reporting. *JAMA*.

[15] Stang A. (2010). Critical Evaluation of the Newcastle-Ottawa Scale for the Assessment of the Quality of Nonrandomized Studies in Meta-Analyses. *European Journal of Epidemiology*.

[16] Zeng X., Zhang Y., Kwong J. S. (2015). The Methodological Quality Assessment Tools for Preclinical and Clinical Studies, Systematic Review and Meta-Analysis, and Clinical Practice Guideline: A Systematic Review. *Journal of Evidence-Based Medicine*.

[17] Zhang X., Tang J., Wang Y. (2024). Visual Environment in Schools and Student Depressive Symptoms: Insights From a Prospective Study Across Multiple Cities in Eastern China. *Environmental Research*.

[18] Zhang X., Du W., Wang Y., Yang W., Wang X., Yang J. (2023). A Multi-Center Cross-Sectional Study on Visual Impairment and Depression Among Students - Jiangsu Province, China, 2017-2022. *China CDC Weekly*.

[19] Zhu X., Meng J., Han C. (2023). CCL2-Mediated Inflammatory Pathogenesis Underlies High Myopia-Related Anxiety. *Cell Discovery*.

[20] Huang J., Dang H., Cai Y., Liu J., Chen Q. (2022). Myopia and Depression Among Middle School Students in China—Is There a Mediating Role for Wearing Eyeglasses?. *International Journal of Environmental Research and Public Health*.

[21] Yu Y., Yan W., Yu J., Xu Y., Wang D., Wang Y. (2022). Prevalence and Associated Factors of Complains on Depression, Anxiety, and Stress in University Students: An Extensive Population-Based Survey in China. *Frontiers in Psychology*.

[22] Zhang H., Gao H., Zhu Y. (2021). Relationship Between Myopia and Other Risk Factors With Anxiety and Depression Among Chinese University Freshmen During the COVID-19 Pandemic. *Frontiers in Public Health*.

[23] Wu Y., Ma Q., Sun H. P., Xu Y., Niu M. E., Pan C. W. (2017). Myopia and Depressive Symptoms Among Older Chinese Adults. *PLoS ONE*.

[24] Łazarczyk J. B., Urban B., Konarzewska B. (2016). The Differences in Level of Trait Anxiety Among Girls and Boys Aged 13–17 Years With Myopia and Emmetropia. *BMC Ophthalmology*.

[25] Ong S. Y., Ikram M. K., Haaland B. A. (2013). Myopia and Cognitive Dysfunction: The Singapore Malay Eye Study. *Investigative Opthalmology and Visual Science*.

[26] van de Berg R., Dirani M., Chen C. Y., Haslam N., Baird P. N. (2008). Myopia and Personality: The Genes in Myopia (GEM) Personality Study. *Investigative Opthalmology and Visual Science*.

[27] Lopez-Hernandez A. E., Miquel-Lopez C., Garcia-Medina J. J., Garcia-Ayuso D. (2024). Impact of Stimulant Treatment On Refractive Errors and Pupil Diameter in Attention Deficit Hyperactivity Disorder. *Acta Ophthalmologica*.

[28] Wu C. S., Tsai T. H., Chen W. L., Tsai H. J., Chien Y. L. (2023). Ophthalmologic Diagnoses in Youths with Autism Spectrum Disorder: Prevalence and Clinical Correlates. *Autism Research*.

[29] Reimelt C., Wolff N., Hölling H., Mogwitz S., Ehrlich S., Roessner V. (2021). The Underestimated Role of Refractive Error (Hyperopia, Myopia, and Astigmatism) and Strabismus in Children With ADHD. *Journal of Attention Disorders*.

[30] Anketell P. M., Saunders K. J., Gallagher S., Bailey C., Little J. A. (2016). Profile of Refractive Errors in European Caucasian Children With Autistic Spectrum Disorder; Increased Prevalence and Magnitude of Astigmatism. *Ophthalmic and Physiological Optics*.

[31] Akinci A., Oner O., Bozkurt O. H., Guven A., Degerliyurt A., Munir K. (2008). Refractive Errors and Ocular Findings in Children With Intellectual Disability: A Controlled Study. *Journal of American Association for Pediatric Ophthalmology and Strabismus*.

[32] Foreman J., Salim A. T., Praveen A. (2021). Association Between Digital Smart Device Use and Myopia: A Systematic Review and Meta-Analysis. *The Lancet Digital Health*.

[33] Guan H., Wang H., Du K. (2018). The Effect of Providing Free Eyeglasses on Children’s Mental Health Outcomes in China: A Cluster-Randomized Controlled Trial. *International Journal of Environmental Research and Public Health*.

[34] Biswas S., El Kareh A., Qureshi M. (2024). The Influence of the Environment and Lifestyle on Myopia. *Journal of Physiological Anthropology*.

[35] Zhang Y., Lv Q., Yin Y. (2024). Research in China About the Biological Mechanisms that Potentially Link Socioenvironmental Changes and Mental Health: A Scoping Review. *The Lancet Regional Health-Western Pacific*.

[36] Dong X., Li J. Y., Dong D. L. (2024). Association of Sleep Traits With Myopia in Children and Adolescents: A Meta-Analysis and Mendelian Randomization Study. *Preventive Medicine*.

[37] Marino C., Andrade B., Campisi S. C. (2021). Association Between Disturbed Sleep and Depression in Children and Youths: A Systematic Review and Meta-Analysis of Cohort Studies. *JAMA Network Open*.

[38] Wright A., Fisher P. L., Baker N., O’Rourke L., Cherry M. G. (2021). Depression and Anxiety in Chronic Fatigue Syndrome: A Systematic Review. *Journal of Psychosomatic Research*.

[39] Khalid K., Padda J., Pokhriyal S. (2021). Pseudomyopia and Its Association With Anxiety. *Cureus Journal of Medical Science*.

[40] Seitler B. N. (2009). Separation-Individuation Issues and Castration Anxiety: Their Curious Influence On the Epigenesis of Myopia. *The American Journal of Psychoanalysis*.

[41] Wirz-Justice A., Skene D. J., Münch M. (2021). The Relevance of Daylight for Humans. *Biochemical Pharmacology*.

[42] Lazzerini Ospri L., Prusky G., Hattar S. (2017). Mood, the Circadian System, and Melanopsin Retinal Ganglion Cells. *Annual Review of Neuroscience*.

[43] Du Y., Meng J., He W., Qi J., Lu Y., Zhu X. (2024). Complications of High Myopia: An Update From Clinical Manifestations to Underlying Mechanisms. *Advances in Ophthalmology Practice and Research*.

[44] Puranik N., Song M. (2024). Insight Into the Association Between Slitrk Protein and Neurodevelopmental and Neuropsychiatric Conditions. *Biomolecules*.

[45] Voogelaar M., Tedja M. S., Guggenheim J. A. (2019). IMI – Myopia Genetics Report. *Investigative Opthalmology and Visual Science*.

[46] de Castro-Catala M., Peña E., Kwapil T. R. (2017). Interaction Between FKBP5 Gene and Childhood Trauma On Psychosis, Depression and Anxiety Symptoms in a Non-Clinical Sample. *Psychoneuroendocrinology*.

[47] Gimelli S., Makrythanasis P., Stouder C., Antonarakis S. E., Bottani A., Bena F. (2011). A De Novo 12Q13.11 Microdeletion in a Patient With Severe Mental Retardation, Cleft Palate, and High Myopia. *European Journal of Medical Genetics*.

[48] El Chehadeh S., Aral B., Gigot N. (2010). Search for the Best Indicators for the Presence of a VPS13B Gene Mutation and Confirmation of Diagnostic Criteria in a Series of 34 Patients Genotyped for Suspected Cohen Syndrome. *Journal of Medical Genetics*.

