# Supplementary material for: Bidirectional Relationship Between Myopia and Mental Disorders
Source: Depress Anxiety. 2025 Dec 1;2025:3543589. doi: 10.1155/da/3543589 (PMC12685417; doi:10.1155/da/3543589)
Supplement: Supporting Information 1 — Table S1. Search strategy for identifying studies on the association between mental disorders and myopia. Table S2. The specific results of quality assessment of include articles of the impact of myopia on mental disorders (n = 15). Table S3. The specific results of quality assessment of include articles of the impact of mental disorders on myopia (n = 8). [file 3543589.f1.docx]

**Supplementary Table 1**. Search strategy for identifying studies on the association between mental disorders and myopia.

| Database | Search terms | |  |
| --- | --- | --- | --- |
| PubMed | #1 | (myopia [MeSH Terms]) OR (myopia [Title/Abstract]) OR (refractive error [MeSH Terms]) OR (refractive error [Title/Abstract]) OR nearsightedness ([Title/Abstract]) | 50245 |
|  | #2 | (mental disorders[MeSH Terms]) OR (mental disorders[Title/Abstract]) OR (mental health[Title/Abstract]) OR (psychopathology[Title/Abstract]) OR (emotion[Title/Abstract]) OR (mood[Title/Abstract]) OR (pressure[Title/Abstract]) OR (stress[Title/Abstract]) OR (anxiety[Title/Abstract]) OR (depressed[Title/Abstract]) OR (despondent[Title/Abstract]) OR (depression[Title/Abstract]) OR (interpersonal relationship[Title/Abstract]) OR (bipolar disorder[Title/Abstract]) OR (schizophrenia[Title/Abstract]) OR (post-traumatic stress disorder[Title/Abstract]) OR (obsessive-compulsive disorder[Title/Abstract]) OR (eating disorder[Title/Abstract]) OR (attention-deficit[Title/Abstract]) OR (hyperactivity disorder[Title/Abstract]) OR (autism spectrum disorder[Title/Abstract]) OR (personality[Title/Abstract]) OR (adjustment disorder[Title/Abstract]) OR (somatic symptom disorder[Title/Abstract]) OR (dissociative disorder[Title/Abstract]) OR (neurocognitive disorder[Title/Abstract]) | 4002782 |
|  | #3 | (("2000/01/01"[Date - Publication]: "2024/07/18"[Date - Publication])) | 23949241 |
|  | #1 AND #2 AND #3 | | 3450 |
| Web of Science | #1 | ((AB=("myopia” OR “refractive error” OR “nearsightedness”)) OR (TI=("myopia” OR “refractive error” OR “nearsightedness”))) | 35965 |
|  | #2 | ((AB=("mental disorders” OR “mental health” OR “psychopathology” OR “emotion” OR “mood” OR “pressure” OR “stress” OR “anxiety” OR “depressed” OR “despondent” OR “depression” OR “interpersonal relationship” OR “bipolar disorder” OR “schizophrenia” OR “post-traumatic stress disorder” OR “obsessive-compulsive disorder” OR “eating disorder” OR “attention-deficit” OR “hyperactivity disorder” OR “autism spectrum disorder” OR “personality” OR “adjustment disorder” OR “somatic symptom disorder” OR “dissociative disorder” OR “neurocognitive disorder”)) OR (TI=("mental disorders” OR “mental health” OR “psychopathology” OR “emotion” OR “mood” OR “pressure” OR “stress” OR “anxiety” OR “depressed” OR “despondent” OR “depression” OR “interpersonal relationship” OR “bipolar disorder” OR “schizophrenia” OR “post-traumatic stress disorder” OR “obsessive-compulsive disorder” OR “eating disorder” OR “attention-deficit” OR “hyperactivity disorder” OR “autism spectrum disorder” OR “personality” OR “adjustment disorder” OR “somatic symptom disorder” OR “dissociative disorder” OR “neurocognitive disorder”))) | 6032153 |
|  | #3 | PY=2000-2024 | 54318020 |
|  | #1 AND #2 AND #3 | | 2774 |
| Wiley online library | #1 | Title: "myopia" OR "refractive error" OR "nearsightedness" OR Abstract: "myopia" OR "refractive error" OR "nearsightedness" | 8670 |
|  | #2 | Title: "mental disorders” OR “mental health” OR “psychopathology” OR “emotion” OR “mood” OR “pressure” OR “stress” OR “anxiety” OR “depressed” OR “despondent” OR “depression” OR “interpersonal relationship” OR “bipolar disorder” OR “schizophrenia” OR “post-traumatic stress disorder” OR “obsessive-compulsive disorder” OR “eating disorder” OR “attention-deficit” OR “hyperactivity disorder” OR “autism spectrum disorder” OR “personality” OR “adjustment disorder” OR “somatic symptom disorder” OR “dissociative disorder” OR “neurocognitive disorder” OR Abstract: "mental disorders” OR “mental health” OR “psychopathology” OR “emotion” OR “mood” OR “pressure” OR “stress” OR “anxiety” OR “depressed” OR “despondent” OR “depression” OR “interpersonal relationship” OR “bipolar disorder” OR “schizophrenia” OR “post-traumatic stress disorder” OR “obsessive-compulsive disorder” OR “eating disorder” OR “attention-deficit” OR “hyperactivity disorder” OR “autism spectrum disorder” OR “personality” OR “adjustment disorder” OR “somatic symptom disorder” OR “dissociative disorder” OR “neurocognitive disorder” | 3827398 |
|  | #1 AND #2 AND From 2000 to 2024 | | 3333 |
| Cochrane | #1 | MeSH descriptor: [myopia] explode all trees | 1722 |
|  | #2 | MeSH descriptor: [refractive error] explode all trees | 3084 |
|  | #3 | (nearsightedness):ti,ab,kw | 60 |
|  | #4 | MeSH descriptor: [mental disorders] explode all trees | 107768 |
|  | #5 | (mental health OR psychopathology OR emotion OR mood OR pressure OR stress OR anxiety OR depressed OR despondent OR depression OR interpersonal relationship OR bipolar disorder OR schizophrenia OR post-traumatic stress disorder OR obsessive-compulsive disorder OR eating disorder OR attention-deficit OR hyperactivity disorder OR autism spectrum disorder OR personality OR adjustment disorder OR somatic symptom disorder OR dissociative disorder OR neurocognitive disorder):ti,ab,kw | 447146 |
|  | (#1 OR #2) AND (#3 OR #4) with Cochrane Library publication date from Jan 2000 to Jul 2024 | | 200 |

**Supplementary Table 2**. The specific results of quality assessment of include articles of the impact of myopia on mental disorders (n=15).

| Study | Item 1 | Item 2 | Item 3 | Item 4 | Item 5 | Item 6 | Item 7 | Item 8 | Item 9 | Item 10 | Item 11 | Points | Grade |
| --- | --- | --- | --- | --- | --- | --- | --- | --- | --- | --- | --- | --- | --- |
| **Cohort study (NOS)** | Representativeness of the exposed cohort | Selection of the non exposed cohort | Ascertainment of exposure | Demonstration that outcome of interest was not present at start of study | Comparability of cohorts on the basis of the design or analysis | Assessment of outcome | Was follow-up long enough for outcomes to occur | Adequacy of follow up of cohorts | / | / | / |  |  |
| Xiyan Zhang, 2024 | 1 | 1 | 1 | 1 | 2 | 1 | 1 | 1 | / | / | / | 9 | High |
| Wei-Po Chou, 2023 | 1 | 1 | 1 | 1 | 2 | 1 | 1 | 1 | / | / | / | 9 | High |
| **Cross-sectional study (AHRQ)** | Define the source of information(survey, record review) | List inclusion and exclusion criteria for exposed and unexposed subjects (cases and controls)or refer to previous publications | lndicate time period used for identifying patients | Indicate whether or not subjects were consecutive if not population-based | Indicate if evaluators of subjective components of study were masked to other aspects of the status of the participants | Describe any assessments undertaken for quality assurance purposes (e.g., test/retest of primary outcome measurements) | Explain any patient exclusions from analysis | Describe how confounding was assessed and/or controlled. | lf applicable, explain how missing data were handled in the analysis | Summarize patient response rates and completeness of data collection | Clarify what follow-up, if any, was expected and the percentage of patients for which incomplete data or follow-up was obtained |  |  |
| Itay Nitzan,2024 | 1 | 1 | 1 | 1 | 1 | 1 | 1 | 1 | 0 | 1 | 0 | 9 | High |
| Xiyan Zhang,2023 | 1 | 1 | 1 | 1 | 1 | 1 | 1 | 1 | 1 | 1 | 1 | 11 | High |
| Xiangjia Zhu,2023 | 0 | 0 | 0 | 1 | 1 | 1 | 1 | 1 | 0 | 0 | 0 | 5 | Medium |
| Juerong Huang,2022 | 1 | 1 | 1 | 1 | 1 | 0 | 0 | 1 | 0 | 1 | 0 | 7 | Medium |
| Yanling Yu,2022 | 1 | 1 | 1 | 1 | 1 | 1 | 1 | 1 | 0 | 1 | 0 | 9 | High |
| Hongmei Zhang,2021 | 1 | 1 | 1 | 1 | 1 | 1 | 1 | 1 | 1 | 1 | 0 | 10 | High |
| Kwadwo Owusu Akuffo,2021 | 1 | 1 | 0 | 1 | 1 | 1 | 1 | 1 | 0 | 1 | 0 | 8 | High |
| Qiaoli Li,2020 | 1 | 1 | 1 | 1 | 1 | 1 | 1 | 1 | 0 | 1 | 0 | 9 | High |
| Yin Wu,2017 | 1 | 1 | 1 | 1 | 1 | 0 | 1 | 1 | 1 | 1 | 0 | 9 | High |
| Joanna B Łazarczyk,2016 | 1 | 1 | 1 | 1 | 1 | 1 | 1 | 1 | 0 | 1 | 0 | 9 | High |
| Tau Yokoi,2014 | 1 | 1 | 1 | 1 | 1 | 1 | 1 | 1 | 0 | 1 | 0 | 9 | High |
| Shin-Yeu Ong,2013 | 1 | 1 | 1 | 1 | 1 | 1 | 1 | 1 | 0 | 1 | 0 | 9 | High |
| Robert van de Berg,2008 | 1 | 1 | 0 | 1 | 1 | 1 | 0 | 1 | 0 | 1 | 0 | 7 | Medium |

**Supplementary Table 3** The specific results of quality assessment of include articles of the impact of mental disorders on myopia (n=8).

| Study | Item 1 | Item 2 | Item 3 | Item 4 | Item 5 | Item 6 | Item 7 | Item 8 | Item 9 | Item 10 | Item 11 | Points | Grade |
| --- | --- | --- | --- | --- | --- | --- | --- | --- | --- | --- | --- | --- | --- |
| **Cohort study (NOS)** | Representativeness of the exposed cohort | Selection of the non exposed cohort | Ascertainment of exposure | Demonstration that outcome of interest was not present at start of study | Comparability of cohorts on the basis of the design or analysis | Assessment of outcome | Was follow-up long enough for outcomes to occur | Adequacy of follow up of cohorts | / | / | / |  |  |
| Wei-Po Chou,2023 | 1 | 1 | 1 | 1 | 2 | 1 | 1 | 1 |  |  |  | 9 | High |
| Chi-Shin Wu,2023 | 1 | 1 | 1 | 1 | 2 | 1 | 1 | 1 |  |  |  | 9 | High |
| **Cross-sectional study (AHRQ)** | Define the source of information(survey, record review) | List inclusion and exclusion criteria for exposed and unexposed subjects (cases and controls)or refer to previous publications | lndicate time period used for identifying patients | Indicate whether or not subjects were consecutive if not population-based | Indicate if evaluators of subjective components of study were masked to other aspects of the status of the participants | Describe any assessments undertaken for quality assurance purposes (e.g., test/retest of primary outcome measurements) | Explain any patient exclusions from analysis | Describe how confounding was assessed and/or controlled. | lf applicable, explain how missing data were handled in the analysis | Summarize patient response rates and completeness of data collection | Clarify what follow-up, if any, was expected and the percentage of patients for which incomplete data or follow-up was obtained |  |  |
| A. Eusebio López-Hernández,2024 | 1 | 1 | 0 | 1 | 1 | 1 | 0 | 1 | 0 | 1 | 0 | 7 | Medium |
| Liping Chen,2023 | 1 | 1 | 0 | 1 | 1 | 1 | 0 | 0 | 0 | 1 | 0 | 6 | Medium |
| Ji Liu,2021 | 1 | 1 | 1 | 1 | 1 | 1 | 0 | 0 | 0 | 1 | 0 | 7 | Medium |
| Charlotte Reimelt,2021 | 1 | 1 | 1 | 1 | 1 | 1 | 0 | 1 | 0 | 1 | 0 | 8 | High |
| Pamela M. Anketell,2016 | 1 | 0 | 0 | 1 | 1 | 1 | 0 | 0 | 0 | 1 | 0 | 5 | Medium |
| Arsen Akinci,2008 | 1 | 1 | 0 | 1 | 1 | 0 | 0 | 1 | 0 | 1 | 0 | 6 | Medium |
