# Supplementary material for: Bidirectional Relationship Between Myopia and Mental Disorders
Source: Depress Anxiety. 2025 Dec 1;2025:3543589. doi: 10.1155/da/3543589 (PMC12685417; doi:10.1155/da/3543589)
Supplement: Supporting Information 2 — Figure S1. The sensitivity analyses of 12 articles on myopia affecting the mental disorders. Figure S2. The sensitivity analyses of eight articles on mental disorders affecting myopia. Figure S3. The sensitivity analyses of myopia on mental disorders by sample size. Figure S4. The sensitivity analyses of mental disorders on myopia by sample size. Figure S5. The sensitivity analyses of myopia on mental disorders by age. Figure S6. The analyses of mental disorders on myopia in those age under 30 years. [file 3543589.f2.docx]

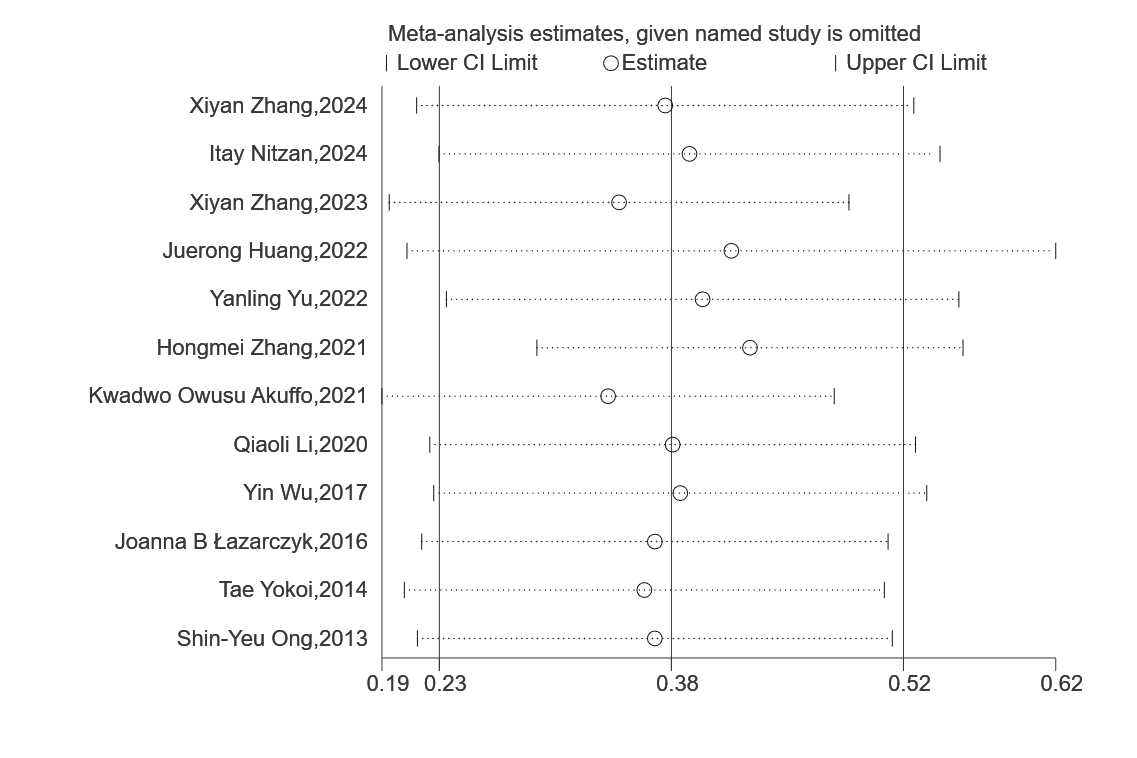
**Supplementary Figure 1** The sensitivity analyses of 12 articles on myopia affecting the mental disorders.

**
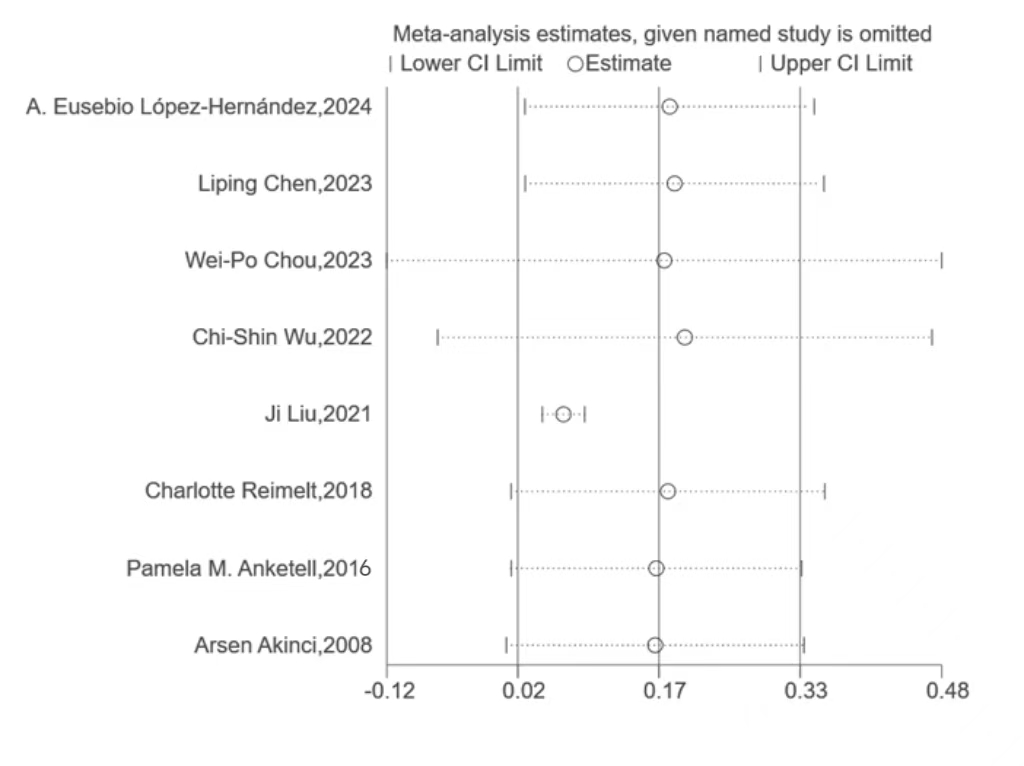
**

**Supplementary Figure 2** The sensitivity analyses of 8 articles on mental disorders affecting myopia.


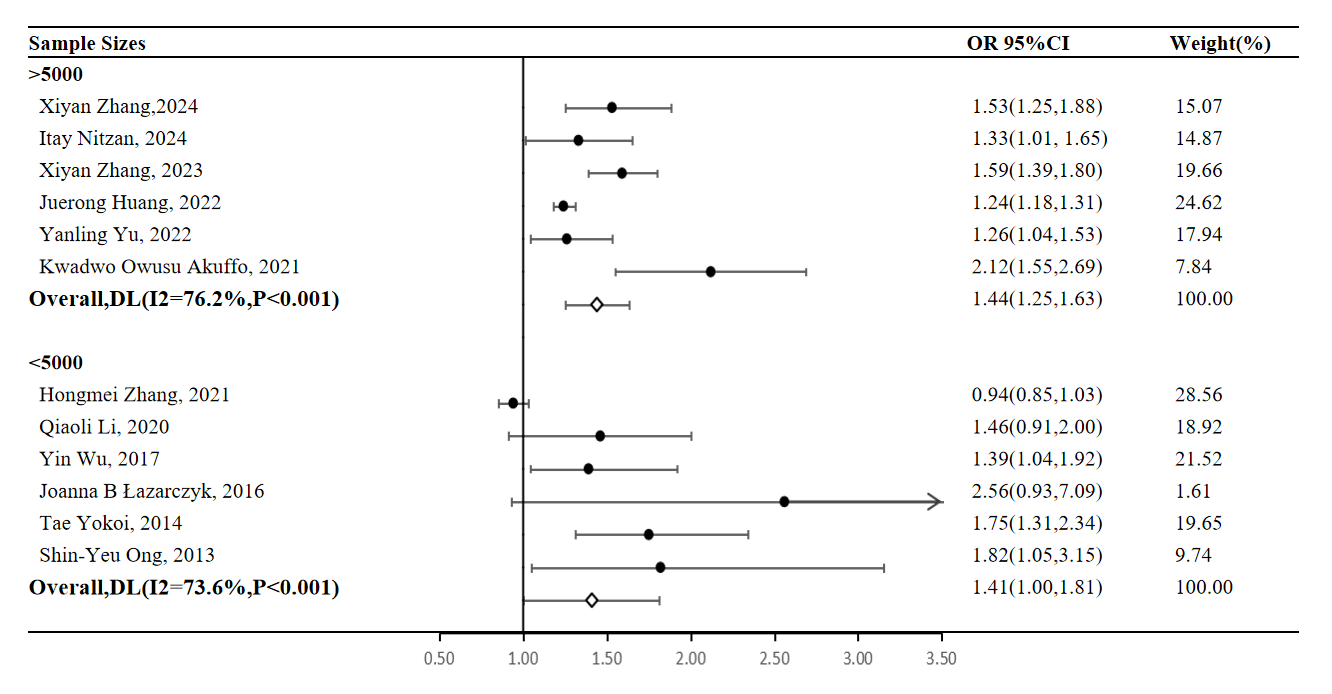
**Supplementary Figure 3** The sensitivity analyses of myopia on mental disorders by sample size.

**
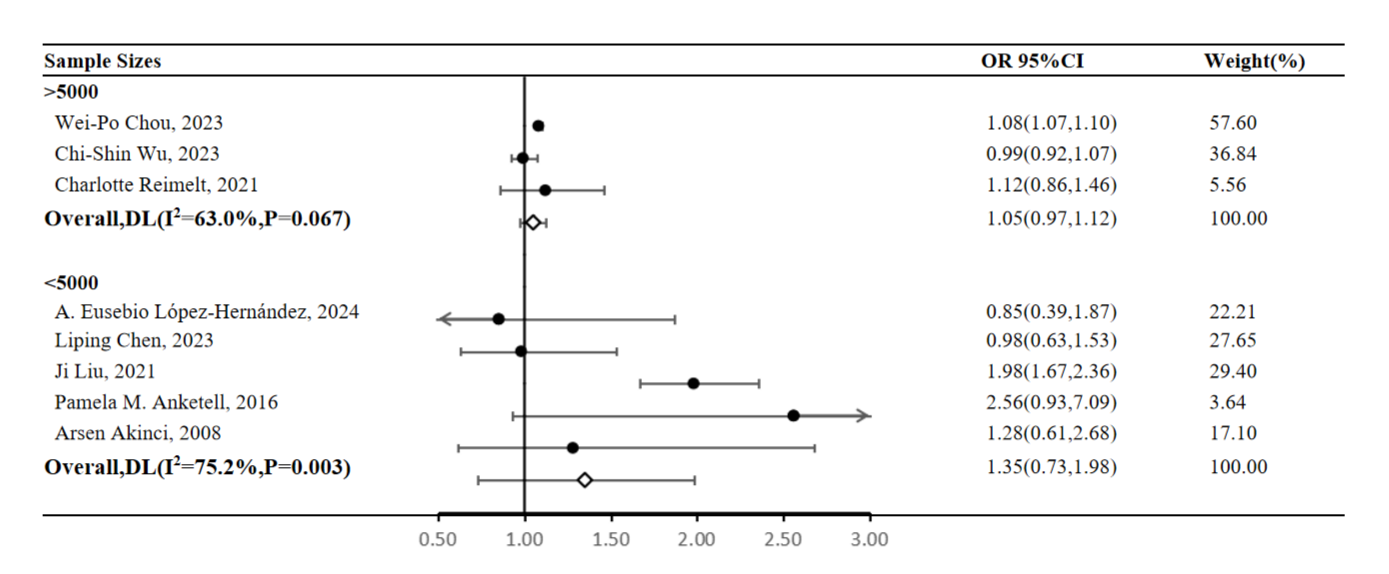
**

**Supplementary Figure 4** The sensitivity analyses of mental disorders on myopia by sample size.


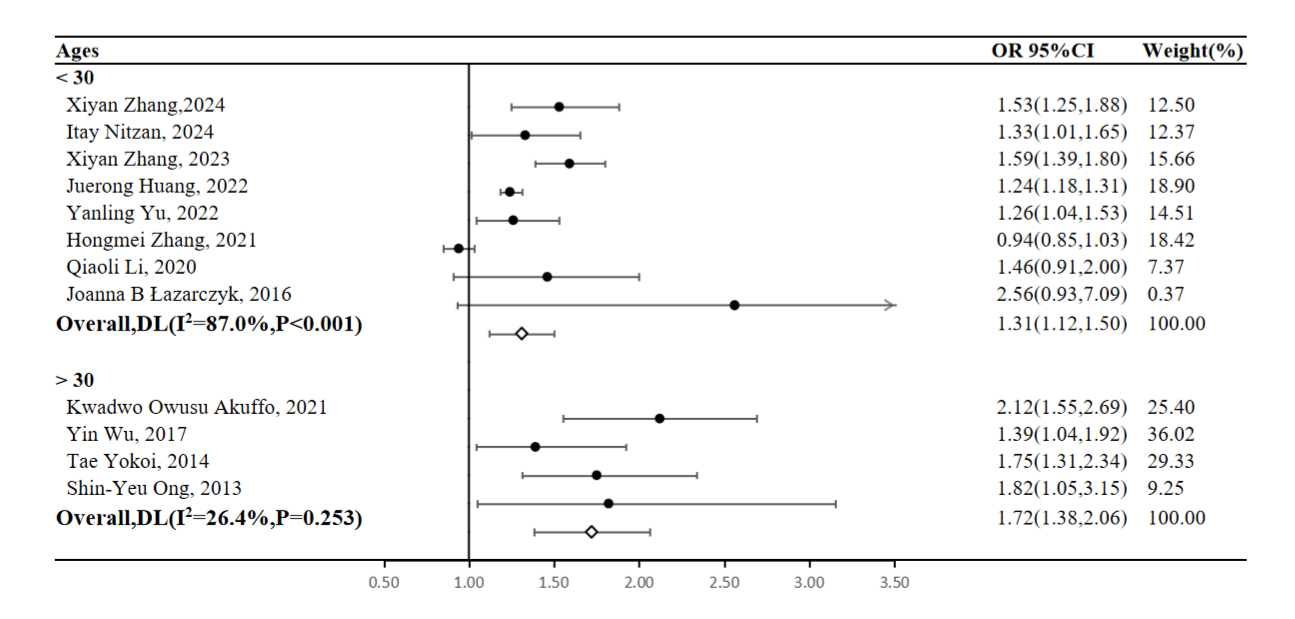
**Supplementary Figure 5** The sensitivity analyses of myopia on mental disorders by age.


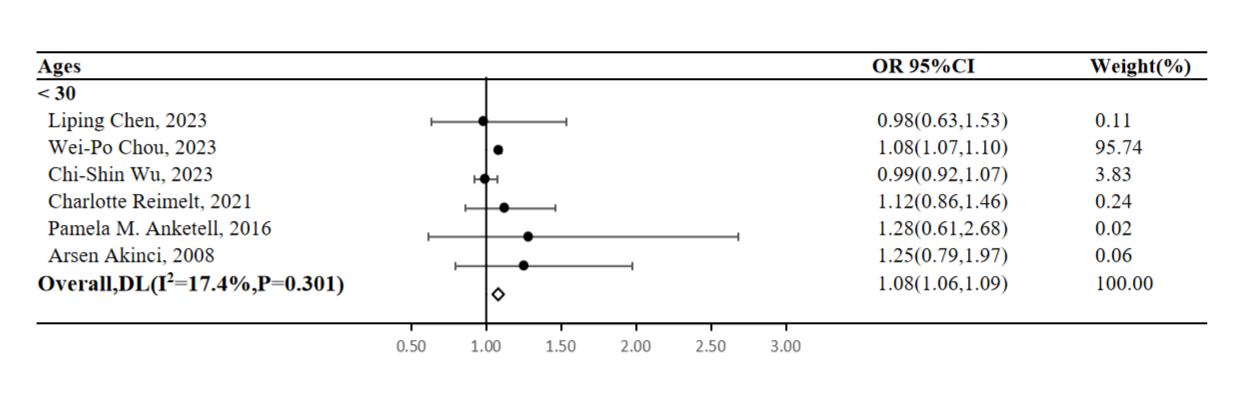
**Supplementary Figure 6** The analyses of mental disorders on myopia in those age under 30 years.
